# SMS text message reminders to improve infant vaccination coverage in Guatemala: A pilot randomized controlled trial

**DOI:** 10.1016/j.vaccine.2016.03.065

**Published:** 2016-05-05

**Authors:** Gretchen J. Domek, Ingrid L. Contreras-Roldan, Sean T. O’Leary, Sheana Bull, Anna Furniss, Allison Kempe, Edwin J. Asturias

**Affiliations:** a Department of Pediatrics, University of Colorado Anschutz Medical Campus, B065, 13123 E. 16th Ave., Aurora, CO 80045, USA; b Center for Global Health, Colorado School of Public Health, Mail Stop A090, 13199 E Montview Blvd, Suite 310, Aurora, CO 80045, USA; c Center for Health Studies, Universidad del Valle de Guatemala, 18 Av. 11-95, Zona 15, Vista Hermosa III, Guatemala City, Guatemala; d Adult and Child Center for Health Outcomes Research and Delivery Science (ACCORDS), University of Colorado Anschutz Medical Campus, Mail Stop F443, 13199 E Montview Blvd, Suite 300, Aurora, CO 80045, USA; e Department of Community and Behavioral Health, Colorado School of Public Health, 13001 E 17th Place, B119, Bldg 500, Rm E3345A, Aurora, CO 80045, USA

**Keywords:** mHealth, Childhood, Immunization, Reminder-recall, SMS, Text messaging

## Abstract

**Background:**

Patient reminder systems are an evidence-based way to improve childhood vaccination rates but are difficult to implement in low- and middle-income countries (LMICs). Short Message Service (SMS) texts may offer a potential low-cost solution, especially in LMICs where mobile phones are becoming more ubiquitous.

**Objective:**

To determine if an SMS-based vaccination reminder system aimed at improving completion of the infant primary immunization series is feasible and acceptable in Guatemala.

**Methods:**

A pilot randomized controlled trial was conducted at two public health clinics in Guatemala City. Infants aged 8–14 weeks presenting for the first dose of the primary immunization series were enrolled in March–April 2013. Participants randomized into the intervention received three SMS reminders one week before the second and third dose. A follow-up acceptability survey was administered to both groups.

**Results:**

The participation rate was 86.8% (321/370); 8 did not own a cell phone and 12 could not use SMS. 96.9% of intervention parents were sent at least one SMS reminder prior to visit 2 and 96.3% prior to visit 3. Both intervention and usual care participants had high rates of vaccine and visit completion, with a non-statistically significant higher percentage of children in the intervention completing both visit 2 (95.0% vs. 90.1%, *p* = .12) and visit 3 (84.4% vs. 80.7%, *p* = .69). More intervention vs. usual care parents agreed that SMS reminders would be helpful for remembering appointments (*p* < .0001), agreed to being interested in receiving future SMS reminders (*p* < .0001), and said that they would be willing to pay for future SMS reminders (*p* = .01).

**Conclusion:**

This proof of concept evaluation showed that a new application of SMS technology is feasible to implement in a LMIC with high user satisfaction. Larger studies with modifications in the SMS system are needed to determine effectiveness (Clinical Trial Registry NCT01663636).

## Highlights

• A novel SMS vaccine reminder platform was created in a LMIC.
• SMS vaccine reminders were proven feasible to implement in a LMIC.
• SMS vaccine reminders were acceptable to use in a LMIC with high user satisfaction.
• SMS vaccine reminders have the potential for widespread scalability at low cost.

## 1 Introduction

Mobile technologies, such as Short Message Service (SMS) texts, have tremendous and untapped potential for disease management in low- and middle-income countries (LMICs). Mobile phone use continues to rapidly increase globally, especially in LMICs where the majority of subscribers now reside [1], [2]. Given its ubiquitous and low-cost nature, mobile telecommunication may be able to break the “digital divide” and be used as an important means to improve health outcomes [3], [4]. Several studies have explored the use of SMS messaging to improve disease prevention in LMICs and have shown promising results [5], [6], [7], [8], [9], [10], [11], [12].

One potential use of SMS involves improving the timeliness and overall completion of the childhood vaccination series. Immunization is one of the most successful and cost-effective public health interventions. However, over 18 million children under the age of one worldwide remain under-immunized and at increased risk for premature death and long-term disability due to vaccine-preventable causes [13]. A major barrier to delivering the infant primary series, especially in LMICs, is the difficulty providers have in communicating with parents regarding the need for and timeliness of these vaccines. Many LMICs do not have functioning preventive health services, making the delivery of immunizations more difficult. There is a need to optimize vaccine supply chains and logistics systems to function effectively and extend the reach of immunizations, especially in difficult to reach populations. Mobile technologies may be able to bridge this gap and transform communication between health care facilities providing immunizations and parents of young children to improve overall vaccination coverage and child health outcomes.

Vaccination rates in Guatemala are well below the United Nations Children's Fund (UNICEF) and World Health Organization's (WHO) Global Immunization Vision and Strategy (GIVS) coverage goal of 90% [14], with only 75% of infants having received the recommended three doses of pentavalent vaccine (diphtheria, tetanus, pertussis, hepatitis B, and Haemophilus influenzae B) by age 12 months in the central Department of Guatemala (which includes Guatemala City) [15]. An SMS reminder platform was developed by the Guatemala Ministry of Public Health and Social Assistance (Ministerio de Salud Pública y Asistencia Social, MSPAS), the Pan American Health Organization (PAHO), and project Optimize to deliver SMS messages to health care workers in order to maintain the quantity and quality of vaccine inventory in a rural area of Guatemala [16]. In collaboration with the MSPAS and PAHO, this platform was modified in the current study to provide SMS reminders to parents. The purpose of this pilot evaluation was to test the feasibility and acceptance of SMS vaccination reminders sent to parents of children presenting for their infant primary immunization series in Guatemala City and to evaluate the potential of a larger scale program to optimize immunization delivery in a LMIC.

## 2 Methods

This evaluation was designed as a pilot randomized controlled trial and was conducted at two public health clinics in Guatemala City serving a publically insured and low-income population. Enrollment occurred between March and April of 2013. Participation was voluntary and parents were not given any incentives. The evaluation was approved by the Colorado Multiple Institutional Review Board, the Universidad del Valle Ethics Committee, and the Guatemala National Ethics Committee of the MSPAS as a quality improvement project.

### 2.1 Participants

Parents of infants between the ages of 8 and 14 weeks presenting for the first dose of the 3-dose infant primary immunization series were eligible if they owned a mobile phone with SMS text messaging capability. At least one parent had to be literate and able to use SMS technology. Parents were excluded if they were under 18 years of age or if their child had a contraindication to vaccination. Study nurses reviewed the protocol with parents, and all participating parents signed a consent for study inclusion and the release of their child's vaccination records. Participants were allocated to either an intervention or usual care group using a computer-generated randomization scheme with the investigators being blind to the allocation. Upon unlocking the database, it was noted that a disproportionate number of participants were randomized into the intervention group during the first month and the usual care group during the second month of the enrollment period. Equal numbers of intervention and usual care participants were enrolled at the two clinic sites.

### 2.2 Intervention

The infant primary immunization series in Guatemala consists of vaccine doses at two months of age (visit 1: pentavalent, pneumococcal, poliomyelitis, and rotavirus), four months (visit 2: pentavalent, pneumococcal, poliomyelitis, and rotavirus), and six months (visit 3: pentavalent and poliomyelitis). Pentavalent vaccine in Guatemala is a combination vaccine for diphtheria, tetanus, pertussis (whole-cell), Haemophilus influenza B, and hepatitis B. All patients, including usual care patients, received written reminders in the child's immunization card for the next dose of vaccines at the time of each vaccination based on the usual standard of care at both clinics. Those randomized into the intervention group also received the following SMS text messages at six, four, and two days before the next scheduled date for visits 2 and 3: “Your child [autopopulate child's name] is due on [autopopulate date] at [autopopulate clinic name] for vaccines.” The SMS messages had a third grade Flesch-Kincaid readability level and were translated into Spanish. Automated SMS texts were generated using a customized computer-based software program hosted at the Health Information Management System (Sistema de Información Gerencial de Salud, SIGSA) of the MSPAS. All children were followed for six months, which allowed two months of observation following each expected visit in the primary immunization series.

### 2.3 Data collection

At enrollment (visit 1), all participants completed a baseline demographic survey. Research nurses collected data at visits 2 and 3, including information regarding family mobile phone use, receipt of SMS reminders, and vaccines given. Immunization records were able to be confirmed for children who returned to either of the two study sites. The mobile carrier report from SIGSA included the number and dates of SMS reminders *sent* to each participant but could not verify if the messages were received or read by the parent. A follow-up satisfaction survey using a 5-point Likert scale was administered by a study nurse to all participants during the final study visit. All surveys were pretested by our study group in Guatemala. A study nurse administered surveys in person during the immunization visits and entered results using a hand-held tablet based device.

### 2.4 Statistical analysis

The primary outcomes for this study were the proportion of parents who were sent SMS messages and parental satisfaction with the intervention. Secondary outcomes included completion of the infant immunization series and the rate of completion by visit and by antigen. Chi-square, Mantel–Haenszel, Fisher's Exact, Wilcoxon Rank Sum, and logistic regression were used to test for differences between the intervention and the usual care group. Both per protocol and intention to treat analyses were done. All analyses were conducted using SAS (SAS 9.3, SAS Institute, Cary, NC).

## 3 Results

### 3.1 Study population

**Fig. 1:**
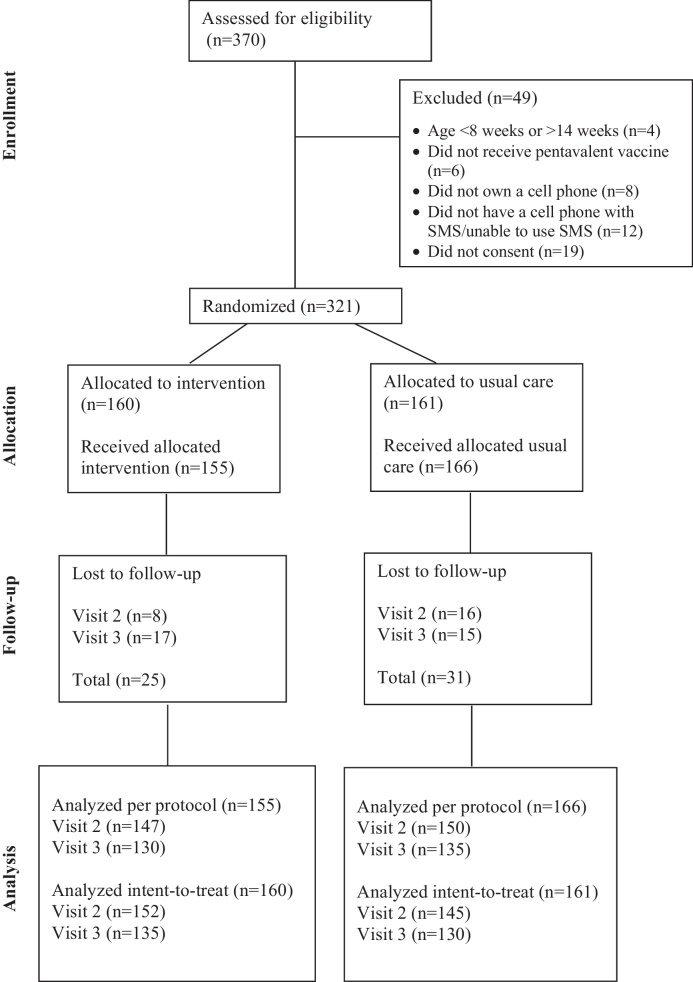
CONSORT diagram.

Of the 370 children screened for eligibility, 321 (86.8%) infants were enrolled in the study (Fig. 1). Forty-nine children were determined ineligible due to older or younger age (*n* = 4), not receiving the child's first pentavalent vaccine (*n* = 6), parent not owning a cell phone (*n* = 8), parent owning a cell phone that does not have SMS capability or unable to use SMS technology (*n* = 12), or parent not giving consent (*n* = 19). Of those patients screened for eligibility, 94.6% (*n* = 350) of parents owned a cell phone and were able to use SMS on their phone. Once enrolled, participants were allocated to the intervention (*n* = 160) or usual care (*n* = 161) group. Parents in the usual care group had significantly higher income with more fathers working; otherwise, there were no significant differences between the baseline demographics of intervention and usual care children and their parents (Table 1). Twenty-four patients (7.5%) were lost to follow-up after the first visit and 32 patients (10.0%) after the second visit for a total of 56 patients (17.4%) lost to follow-up during the study.

### 3.2 Text message delivery

According to the mobile carrier report, a total of 373 SMS reminder messages were sent prior to visit 2 and 409 prior to visit 3; 96.9% (*n* = 155) of intervention parents were sent at least one SMS reminder prior to visit 2 and 96.3% (*n* = 154) prior to visit 3 (Table 2). While a majority of children randomized to the SMS reminder group were sent the intended three reminders (52.5% for visit 2 and 46.9% for visit 3), some parents were sent fewer than three messages (47.5% for visit 2 and 41.3% for visit 3) and other parents were sent four or five messages prior to visit 3 (11.9%). There were challenges with the SMS delivery system that included power outages and delays in recharging the server that either resulted in missed messages or repeat messages being delivered upon reactivation of the delivery platform.

### 3.3 Impact of text message reminders

Per protocol analyses were consistent with intention to treat. Both intervention and usual care groups had high rates of vaccine and visit completion (Table 3). A non-statistically significant higher percentage of children completed each immunization series in the intervention group compared to the usual care group (84.4% vs 80.7% for pentavalent and poliomyelitis, 90.0% vs. 83.2% for pneumococcal, and 91.9% vs. 88.8% for rotavirus). A higher percentage of children in the intervention versus usual care group also completed both visit 2 (95.0% vs. 90.1%) and visit 3 (84.4% vs. 80.7%), although no result reached statistical significance.

### 3.4 Parent satisfaction

**Fig. 2:**
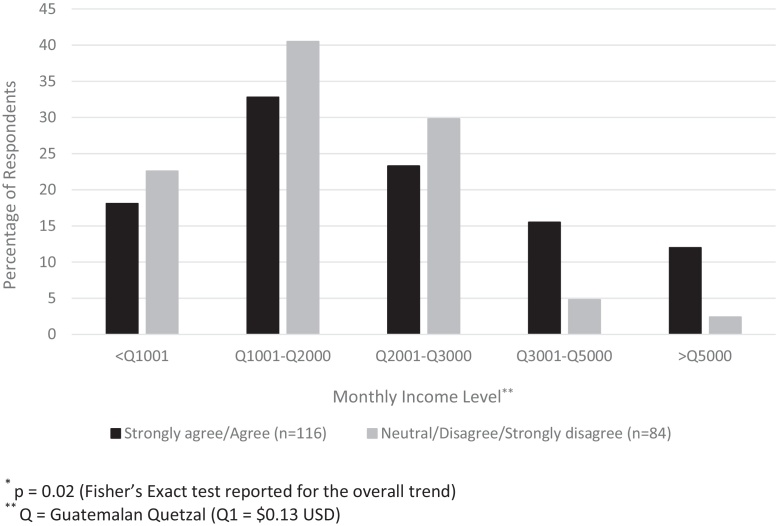
Comparison^*^ of those who agree vs. those who do not agree with the statement ‘Would be willing to pay for text message reminders’ by monthly income level.

Follow-up survey response rates for those completing visit 3 were 83.0% (*n* = 112) for intervention parents and 80.0% (*n* = 104) for usual care parents (Table 4). Of those in the intervention, 78.3% remembered receiving SMS messages. In general, intervention parents had more favorable views toward SMS reminders than those in the usual care group. More intervention parents agreed that SMS reminders would be helpful for remembering appointments compared to usual care parents (*p* < .0001), agreed to being interested in receiving future SMS reminders (*p* < .0001), and said that they would be willing to pay for future SMS reminders (*p* = .01). Participants with a higher income in both groups were more likely to agree to paying for SMS reminders (*p* = .02) (Fig. 2). All intervention parents would recommend SMS reminders to family.

## 4 Discussion

Our results from this pilot evaluation demonstrated that it was feasible and acceptable for parents to use an SMS-based vaccine reminder system in a LMIC, such as Guatemala, with high user satisfaction. Although this pilot was not adequately powered to detect statistically significant differences in a population with already high vaccine coverage rates, the observed point estimate for the difference is consistent with previous literature on reminder–recall systems [17], [18], [19], [20]. Furthermore, the lessons learned during this pilot will help inform future large scale randomized trials. Despite problems with the SMS delivery software noted above, the SMS system proved feasible and worked well enough to deliver at least one SMS to almost every family. Additionally, almost all parents in this low-resource setting had access to a cellular phone with SMS capability and were able to use SMS technology. The results related to acceptance are also encouraging. Parents were willing to provide their cellular number to receive text reminders, with very few parents choosing not to participate. Parents reported high user satisfaction, with almost all parents expressing interest in receiving future SMS reminders and many parents even willing to pay for these reminders. Overall, our data suggest that with a well-functioning system, integration of SMS for vaccine reminders in LMICs would be both feasible and accepted by parents.

Reminder–recall systems that inform when patients are due or overdue for specific immunizations have been tested in the primary care setting of high-income countries and have been shown to effectively improve immunization rates for both children and adults, including postcards, letters, e-mails, and telephone or auto dialer calls [17], [18], [19], [20]. Despite these evidence-based interventions, such methods are being underutilized due to a lack of resources, especially in low-income populations where reminder-recalls have been difficult to implement successfully [21], [22], [23]. Newer and more cost-effective systems are being sought. Vaccine reminders with SMS technology have yielded promising results when used in high-income countries. Studies have shown increased compliance with vaccination schedules in travelers receiving the hepatitis A and B vaccine series [24], pediatric patients receiving influenza [25], [26] and post shortage Haemophilus influenza B [27], adolescent patients receiving MCV4/Tdap [27] and human papillomavirus [28] immunizations, and children receiving their primary immunization series [29] and MMR vaccination [30]. Preliminary studies are promising and suggest that parents may even prefer text message reminders over other forms of communication [31], [32]. Additionally, a recent meta-analysis demonstrated the strong potential of text messaging as an intervention to facilitate healthy behaviors and identified a number of “SMS best practices” consistent across effective studies in several countries, including sending tailored, targeted, and personalized messages [33]. While there is growing evidence to support SMS applications for health promotion, most research has been done in high-income countries [34], and the evaluation of these interventions in LMICs has generally not been done consistently or rigorously [5], [35]. While SMS reminder applications have been tested in LMICs, we are only aware of one other LMIC study using SMS to improve completion of the infant immunizations series [36].

This evaluation had several limitations. As a pilot study we were not adequately powered to assess efficacy; the aforementioned system implementation issues contributed to the potentially limited impact of the program. Even when SMS texts were delivered, the MSPAS platform did not have a reporting feature, so we were only able to verify when a text was sent by the mobile carrier but not if it was received or read by the parent. Thus, we do not know if those parents who did not recall receiving the text messages did not, in fact, receive the reminders or if they forgot. We experienced another limitation when we were unable to track childhood vaccination records outside of the study clinics. The MSPAS is in the process of creating a computerized National Immunization Registry to track childhood vaccinations. However, this registry was not yet functional at the time of our evaluation, and we were only able to confirm immunization records for children who returned to one of our two study sites. This evaluation was also limited by a potential selection bias. Children in both groups had high visit and immunization completion rates, making it more difficult to show an effect. This may be because children were recruited at visit 1 if they presented to the clinic within six weeks of the visit due date, creating a bias for those children already receiving their immunizations on time. As this was a pilot evaluation, it was further limited by a small sample size and was only conducted in one setting, therefore making it less generalizable. The baseline sample was also not equivalent on income and fathers working. While this is not unusual with a small sample size, it is possible that the disproportionate number of participants randomized into the intervention during the first month of the study accounted for this difference in employment and income. There were intermittent shortages of pneumococcal vaccine available in the private sector during our study, which may have resulted in some of these patients attending the public clinics for immunizations.

There are several lessons learned during this pilot that will help inform future large scale randomized trials in LMICs. We suggest having a back-up server and potentially a generator with a detailed protocol to resend messages after outages to ensure that parents in future studies are able to receive the intended SMS reminders. Other studies have implemented bi-directional messaging into their automated SMS delivery programs to allow recipients to confirm receipt of a message. This was not implemented in our study because, while our participants could receive an SMS text without charge, they would have had to pay to send a bi-directional confirmation message. This is an area that could be explored in future studies. It will also be important in the future to demonstrate test message implementation linked to an immunization registry in a LMIC.

This study had several strengths, including very high recruitment and little loss to follow-up relative to other studies. Although not reaching statistical significance, a higher percentage of intervention children completed each vaccine and both immunization visits. This suggests that while the use of mobile phones and text messaging by public health systems still remains in its infancy on a global scale, the potential for this technology to break the barriers between the intended goals of national immunization programs and the ultimate users of vaccines is maturing as a promising opportunity. There is a need for a greater number of studies evaluating SMS with sound methodology and sufficient statistical power that are based in resource-limited settings, especially with regard to large-scale implementation.

## 5 Conclusion

Given the ubiquitous nature and relative affordability of mobile phones, these devices have enormous potential to improve health outcomes in LMICs by reaching large populations at low cost. Vaccine coverage in many LMICs remains below acceptable levels, placing children at risk for unnecessary morbidity and mortality. Although patient reminder systems are an evidence-based way to improve childhood vaccination rates, they have proven difficult to implement in low-resource settings. SMS texts offer a possible low cost solution with the advantageous potential for scalability at little to no increased cost. This proof of concept evaluation showed that a new application of SMS technology could be widely implemented in a LMIC with few parents not able to use SMS and high parent satisfaction. Larger studies with modifications in the SMS system are needed to determine effectiveness.

## Figures and Tables

**Table 1:** Baseline demographic characteristics of entire study population and by study group.

Characteristic	Study population (*n* = 321), % (*n*)	Intervention (*n* = 160), % (*n*)	Usual care (*n* = 161), % (*n*)	*p*-value (Intervention vs usual care)
Child's age				
Age (weeks) at visit 1 [mean (med)]	9.5 (9.0)	9.7 (9.1)	9.4 (9.0)	0.44^a^

Child's gender				
Male	48.4 (155)	52.5 (84)	44.4 (71)	0.15
Female	51.6 (165)	47.5 (76)	55.6 (89)	

Birth weight <6 pounds				
Yes	16.2 (52)	18.1 (29)	14.3 (23)	0.35
No	83.8 (269)	81.9 (131)	85.7 (138)	

Distance to clinic <16 km (10 miles)				
Yes	98.8 (317)	98.1 (157)	99.4 (160)	0.37
No	1.2 (4)	1.9 (3)	0.6 (1)	

Number of siblings				
0 siblings	53.8 (172)	57.5 (92)	50.0 (80)	0.17
1–3 siblings	44.1 (141)	39.4 (63)	48.8 (78)	
4+ siblings	2.2 (7)	3.1 (5)	1.3 (2)	

Monthly income^b^				
<Q1001	18.3 (54)	27.3 (42)	8.5 (12)	<.0001
Q1001–Q2000	35.6 (105)	46.1 (71)	24.1 (34)	
Q2001–Q3000	27.1 (80)	17.5 (27)	37.6 (53)	
>Q3000	19.0 (56)	9.1 (14)	29.8 (42)	

Mother's education				
Primary	34.1 (109)	36.9 (59)	31.3 (50)	0.35
Secondary	35.0 (112)	31.3 (50)	38.8 (62)	
College	30.9 (99)	31.9 (51)	30.0 (48)	

Father's education				
Primary	35.3 (113)	36.9 (59)	33.8 (54)	0.06
Secondary	35.3 (113)	29.4 (47)	41.3 (66)	
College	29.4 (94)	33.8 (54)	25.0 (40)	

Mother has cell phone				
Yes	98.7 (301)	98.7 (148)	98.7 (153)	>0.99
No	1.3 (4)	1.3 (2)	1.3 (2)	

Father has cell phone				
Yes	97.7 (85)	100.0 (39)	95.8 (46)	0.50
No	2.3 (2)	0.0 (0)	4.2 (2)	

Mother works				
Yes	24.1 (77)	26.3 (42)	21.9 (35)	0.36
No	75.9 (243)	73.8 (118)	78.1 (125)	

Father works				
Yes	98.3 (281)	96.5 (136)	100.0 (145)	0.03
No	1.7 (5)	3.5 (5)	0.0 (0)	

a Wilcoxon Rank Sum test.

b *Q* = Guatemalan Quetzal (Q1 = $0.13 USD).

**Table 2:** Number (%) of intervention parents sent text messages (mobile carrier report).

Number of text messages sent	Visit 2 (*n* = 160) *n* (%)	Visit 3 (*n* = 160) *n* (%)
0	5 (3.1)	6 (3.8)
1	21 (13.1)	20 (12.5)
2	50 (31.3)	40 (25.0)
3	84 (52.5)	75 (46.9)
4	–	11 (6.9)
5	–	8 (5.0)

**Table 3:** Study demographics and study outcomes by Intention to Treat (ITT) study groups.

Measure	Intervention (*n* = 160)	Usual care (*n* = 161)	*p*-value^a^
Vaccinations			
Completing Pentavalent (3 doses) [% (*n*)]	84.4 (135)	80.7 (130)	0.69
Completing Pneumococcal (2 doses) [% (*n*)]	90.0 (144)	83.2 (134)	0.34
Completing Poliomyelitis (3 doses) [% (*n*)]	84.4 (135)	80.7 (130)	0.69
Completing Rotavirus (2 doses) [% (*n*)]	91.9 (147)	88.8 (143)	0.44
Completing all vaccinations [% (*n*)]	81.3 (130)	75.8 (122)	0.94
Completing *any* antigen [% (*n*)]	93.1 (149)	88.8 (143)	0.22

Visits			
Completing visit 2 [% (*n*)]	95.0 (152)	90.1 (145)	0.12
Completing visit 3 [% (*n*)]	84.4 (135)	80.7 (130)	0.69
Age (days) at visit 2 [mean (med)]	134.9 (127.0)	131.3 (126.0)	0.52
Age (days) at visit 3 [mean (med)]	195.4 (188.0)	193.9 (187.5)	0.72

a All *p*-values are adjusted for income.

**Table 4:** Attitudes regarding the reminder intervention.

Question^a^	Intervention (*n* = 112) % (*n*)	Usual care (*n* = 104) % (*n*)	*p*-value
Text message reminders would be helpful for remembering appointments			
Strongly agree	26.8 (30)	2.9 (3)	<0.0001^b^
Agree	71.4 (80)	88.6 (93)	
Neutral	1.8 (2)	7.6 (8)	
Disagree	0	1.0 (1)	

Interested in receiving text message reminders in the future			
Strongly agree	31.3 (35)	2.9 (3)	<0.0001^b^
Agree	67.9 (76)	93.3 (98)	
Neutral	0	1.9 (2)	
Disagree	0.9 (1)	1.9 (2)	

Would be willing to pay for text message reminders			
Strongly agree	9.8 (11)	1.0 (1)	0.01^c^
Agree	57.7 (59)	48.6 (51)	
Neutral	29.5 (33)	39.0 (41)	
Disagree	8.0 (9)	11.4 (12)	

a No respondents answered “Strongly Disagree” to any of the three questions; therefore, that response line has been removed from the table.

b Fisher's Exact test.

c Mantel–Haenszel Chi-square test.
